# Invasive pleural malignant mesothelioma with rib destruction and concurrent osteosarcoma in a dog

**DOI:** 10.1186/s13028-015-0176-1

**Published:** 2015-12-02

**Authors:** Morena Di Tommaso, Francesca Rocconi, Giuseppe Marruchella, Anna Rita D’Angelo, Stefano Masci, Domenico Santori, Carla Civitella, Alessia Luciani, Andrea Boari

**Affiliations:** Faculty of Veterinary Medicine, University of Teramo, Località Piano D’Accio, 64100 Teramo, Italy; Istituto Zooprofilattico Sperimentale dell’Abruzzo e Molise “G. Caporale”, Via Campo Boario, 64100 Teramo, Italy

**Keywords:** Chest wall, Bone lysis, Mesothelioma, Osteosarcoma

## Abstract

A 7-year-old Dachshund was clinically examined because of a 10-day history of lameness in the left hind limb. On the basis of radiological and cytological findings, an osteosarcoma of the left acetabular region was suspected. The dog underwent a hemipelvectomy and osteosarcoma was diagnosed by subsequent histopathological examination. An immovable subcutaneous mass was noted on the left chest wall during the physical examination and non-septic neutrophilic inflammation was diagnosed by cytology. Forty days later, the dog showed signs of respiratory distress with an in-diameter increase of the subcutaneous mass up to 4 cm. Thoracic radiography and ultrasonography revealed pleural effusion and a lytic process in the fourth left rib. Furthermore, ultrasound examination revealed a mixed echogenic mobile structure with a diameter of around 2 cm floating within the pleural fluid of the left hemithorax close to the pericardium. The dog underwent surgery for an *en bloc* resection of the subcutaneous mass together with the fourth rib and the parietal pleura. Moreover, the left altered lung lobe, corresponding to the mobile structure detected by ultrasound, was removed. Based on cytological, histopathological, and immunohistochemical examinations, an invasive epithelioid pleural malignant mesothelioma was diagnosed.

## Background

Malignant mesothelioma is an uncommon tumor arising mostly from the serosal surface of the pleural or peritoneal cavity [1]. In most human patients, pleural mesothelioma is a locally advanced tumor and the invasion of the thoracic wall with rib involvement has been reported [2, 3]. A locally invasive nature of malignant mesotheliomas has rarely been reported in dogs, while involvement of ribs has not been reported at all. Only one report describing tumor infiltration of the submesothelial muscle layer in a dog has been published [4].

The simultaneous occurrence of a mesothelioma along with other primary malignancies has been reported repeatedly in humans, whereas no such cases have been reported in dogs [5]. Pleural mesotheliomas have been described mainly in association with carcinomas [5], but a mesothelioma associated with a synchronous osteosarcoma has never been described in neither humans nor dogs.

The present case represents the first report of a diffuse pleural mesothelioma directly invading the thoracic wall in a dog having a concurrent osteosarcoma.

## Case presentation

A 7-year-old male Dachshund weighing 7.6 kg was admitted to the Veterinary Teaching Hospital, University of Teramo, Italy, with a 10-day history of lameness in the left hind limb. During physical examination, the dog showed a grade 4 lameness on a 1–4 scale and strong pain upon palpation of the proximal portion of the left hind limb. Furthermore, an immovable subcutaneous mass measuring around 1.5 cm in diameter was noted on the left chest wall. The complete blood count, serum chemistry profile, urinalysis, and hemostatic profile were unremarkable.

A radiographic examination of the pelvis and hind limbs showed osteolytic lesions in the left ischiatic bone of the acetabular region and subtle thickening of the cortices of the caudal acetabular rim.

A fine needle aspirate (FNA) cytology of the acetabular lesions was done and showed moderate cellularity with a mixture of individual and multinucleated cells. The individual cells were round to oval and had variably shaped nuclei, prominent and multiple nucleoli, and dark blue vacuolated cytoplasma containing fine pink granules. The multinucleated cells were regarded as being osteoclasts. Furthermore, a pink intercellular matrix was found. Based on these findings, an osteosarcoma was diagnosed. A FNA cytology of the subcutaneous mass on the thoracic wall showed several neutrophils in a debris but without visible microorganisms consistent with a non-septic neutrophilic inflammation. A bacteriological examination was done but bacteria were not cultured. Thoracic radiography and abdominal ultrasound were unremarkable for metastatic lesions.

The dog underwent a hemipelvectomy and a histopathological examination of the excised tissue was performed. Microscopically, a neoplastic lesion consisting primarily of polyhedral cells with moderate nuclear pleomorphism was observed. The mitotic index, expressed as the mean value of mitoses/10 high power fields (total magnification, ×400), was 1.1. The neoplasm was infiltrative and gave rise to a small amount of osteoid matrix. The findings were consistent with a productive osteoblastic osteosarcoma. The owner declined any further treatment.

**Fig. 1:**
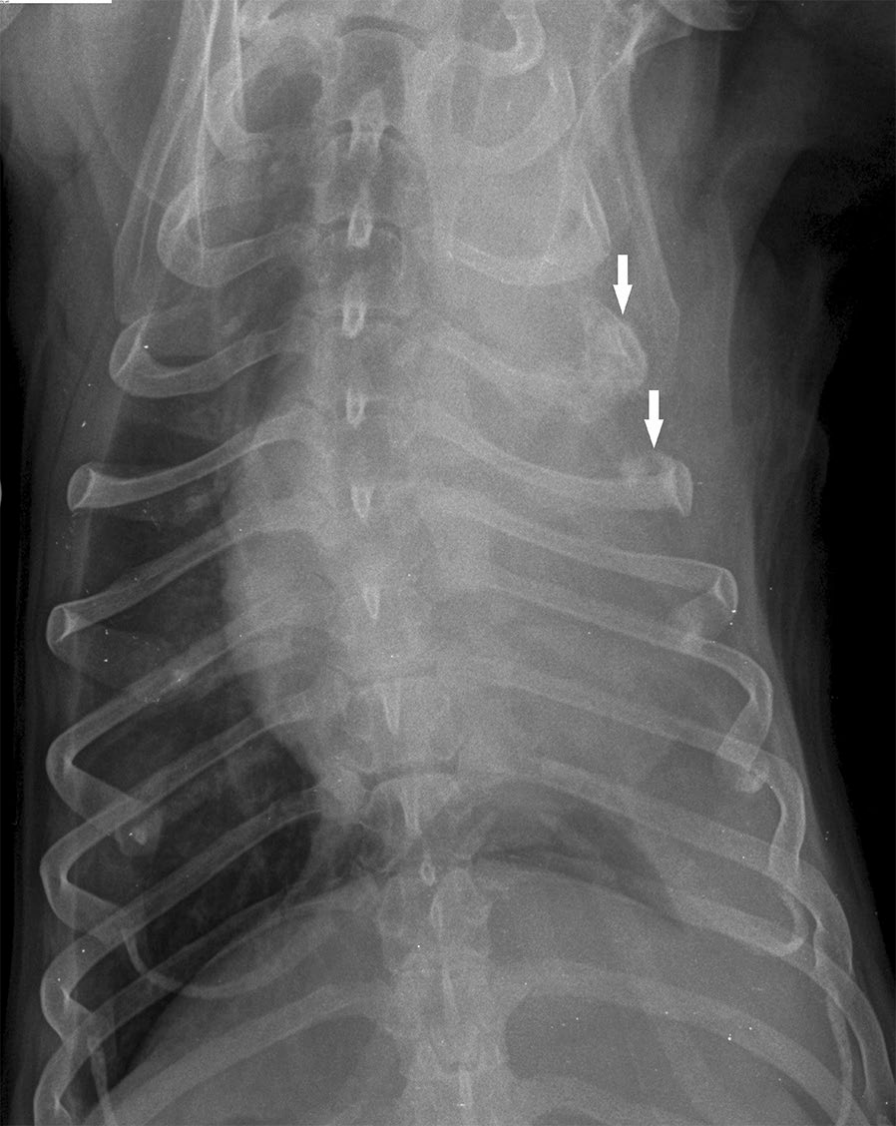
Radiographic examination of the left hemithorax on a ventrodorsal projection. Presence of an osteolytic lesion with periosteal reaction on the fourth and fifth ribs (*arrows*) and pleural effusion

Forty days after this, the dog showed signs of respiratory distress. The subcutaneous mass on the thoracic wall had increased in diameter up to 4 cm. A thoracic radiography revealed small to moderate amounts of pleural effusion in the left hemithorax. A pleural reflection was visible on the right side between the medial and caudal lobes. Furthermore, the fourth left rib showed an expanded contour with thinning of the cortices, bone destruction, and an interrupted periosteal reaction. A mild periosteal reaction was noted on the fifth left rib. Soft tissue swelling was remarkable superficially between the third and sixth rib of the left hemithorax (Fig. 1).

**Fig. 2:**
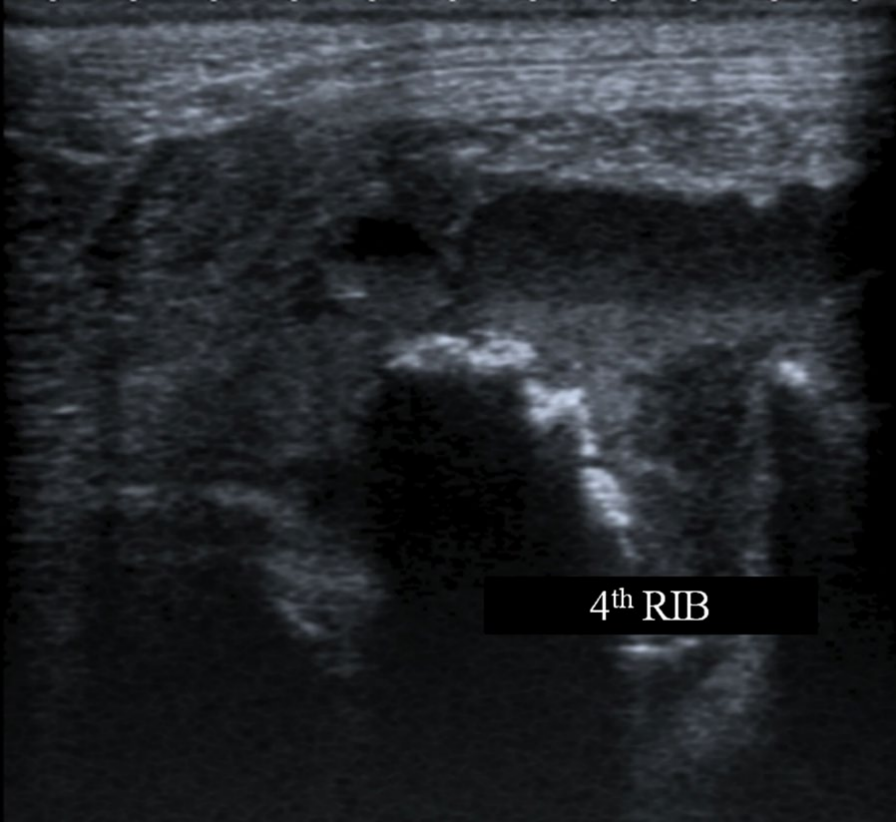
Ultrasonographic examination of the left fourth rib. Irregularly thickened cortical margins associated with hypoechoic areas

An ultrasound examination of the subcutaneous mass showed a hypoechoic cavitating lesion of 3 cm in diameter with a corpuscular content adherent to the fourth rib. Furthermore, the margins of the fourth rib appeared irregularly thickened with hypoechoic areas being consistent with a lytic process (Fig. 2). An ultrasonography of the thorax revealed a moderate pleural effusion, irregularly thickened parietal pleura, and a mixed echogenic mobile structure of around 2 cm in diameter, floating in the pleural fluid of the left hemithorax close to the pericardium. On the basis of the ultrasound examination, consolidated or atelectatic lung tissue, primary or metastatic lung tumor, lung lobe torsion, and thoracic mass were considered.

The FNA cytology of the subcutaneous mass was repeated. The cytological pattern was similar to the previous FNA cytology, whereas cytology of the bone aspirate of the fourth rib was non-diagnostic.

**Fig. 3:**
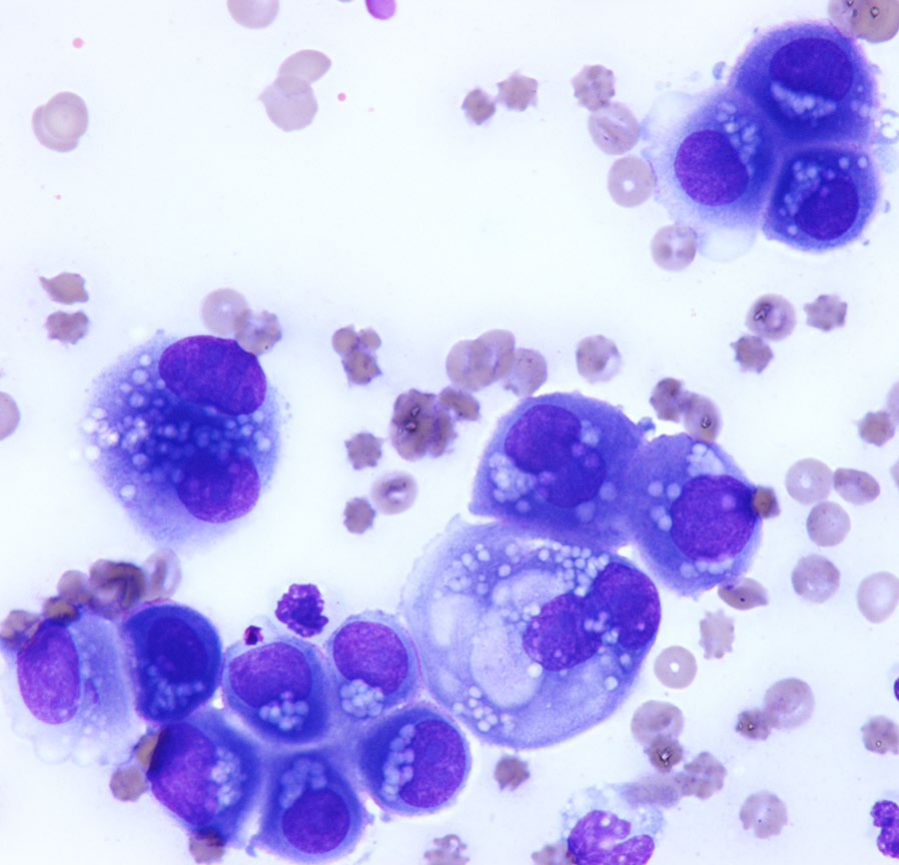
Cytology of pleural fluid. Clumps of neoplastic cells with anisocytosis, anisokaryosis, binucleations, single to multiple prominent and variably shaped nucleoli, and cytoplasmic vacuoli. The presence of a thick brush border and the “mesothelial slits” suggest a mesothelial origin of cells. May-Grünwald Giemsa staining, Obj ×60

The nucleated cell count in the thoracic fluid was 29.7 × 10^3^ cells/µl, and the protein concentration was 5.2 g/dl. Direct smears showed large clumps of cells with “mesothelial slits”, anisocytosis, aniso-macrokaryosis, multinucleation, and single to multiple prominent and variably shaped nucleoli. Several cells presented cytoplasmic vacuoli and the formation of a thick brush border. A moderate number of neutrophils was also present. The cytological pattern suggested a mesothelioma (Fig. 3).

The dog underwent surgery for an *en bloc* resection of the subcutaneous mass together with the fourth rib and the parietal pleura. Furthermore, the left altered lung lobe was removed.

**Fig. 4:**
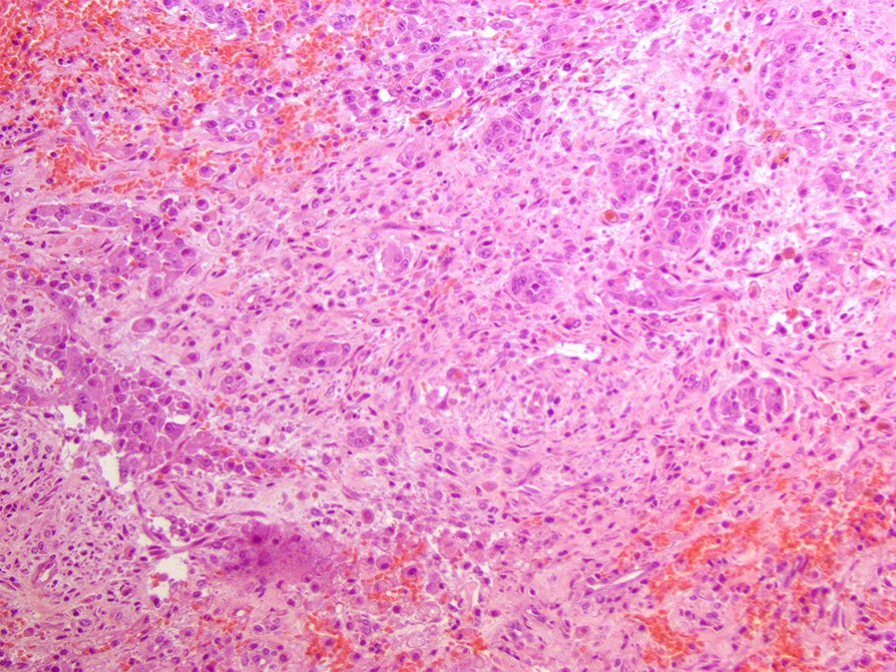
Photomicrograph of the mesothelioma. Several clusters of epithelioid cells, sometimes arranged as tubules and glandular-like structures, are seen embedded within a prominent fibrotic reaction. Hemorrhagic foci are also observed throughout the neoplastic lesion. Hematoxylin and eosin, Obj ×40

**Fig. 5:**
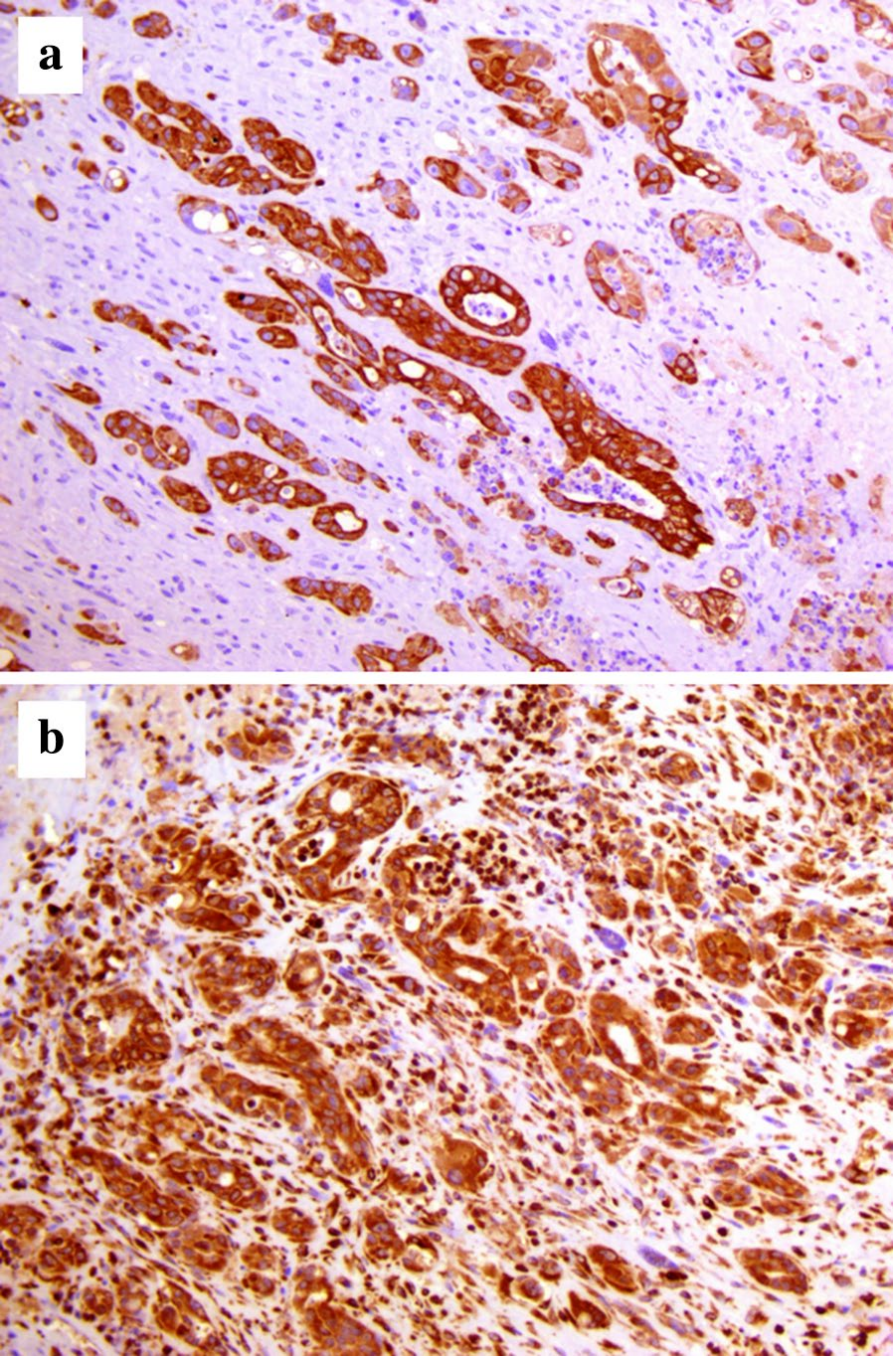
Immunohistochemical staining of the mesothelioma. Neoplastic cells show a strong and specific immunoreactivity for **a** pancytokeratin and **b** vimentin. Mayer’s hematoxylin counterstain, Obj ×40

The excised tissues were fixed in 10 % neutral buffered formalin, embedded in paraffin, and routinely processed for histopathological examination (hematoxylin and eosin stain). Microscopically, a neoplastic lesion was observed. The tumor infiltrated all thoracic wall layers including the bone cortex and the rib periosteum, and showed a multifocal pattern in the lung tissue. The neoplasm consisted primarily of epithelioid cells that were arranged as cords or nests or showed a “gland-like” tubular pattern. Neoplastic cells contained abundant eosinophilic, occasionally vacuolated, cytoplasm and were interspersed within abundant connective tissue (Fig. 4). Mitotic figures were observed with a mitotic index of 1.7. A number of neoplastic emboli were found within the pulmonary blood vessels. In addition, large foci of purulent inflammation were scattered throughout the neoplasm. Immunohistochemical investigations for pancytokeratin (clone AE1/AE3, final dilution 1:500 Dako; Glostrup, Denmark) and vimentin (clone V9, final dilution 1:800 Dako) were carried out on selected tissue sections. Epithelioid neoplastic cells showed a strong and specific immunoreactivity for pancytokeratin and vimentin (Fig. 5). Consequently, an invasive epithelioid pleural malignant mesothelioma was diagnosed.

The dog died from complications arising after surgery. The owner declined a necropsy.

Though mesothelioma is the most common primary tumor of serosal membranes, it is a rare neoplasm both in humans and dogs [1, 5]. Most cases of pleural mesothelioma occur as diffuse tumors covering the surfaces of the body cavity, whereas the localized forms are rare [6, 7].

Mesotheliomas are considered malignant due to their ability to spread by implantation, but they rarely metastasize in dogs [1, 8]. Malignant pleural mesothelioma rarely metastasizes to distant sites in humans, whereas a locally advanced disease appears in most patients; the invasion of the chest wall with rib destruction has also reported [2, 3]. Indeed, according to the International Mesothelioma Interest Group Staging System of pleural mesothelioma, this condition, with or without rib destruction is described in stages III and IV [9]. A review of the veterinary literature revealed only two canine cases with the chest wall being invaded with a mesothelioma. Reggetti et al. [4] described a case of a diffuse mesothelioma that infiltrated the muscle layer underlying the pleura but, unlike what we found, both ribs and subcutaneous tissue were not involved. Liptak and Brebner [10] reported two subcutaneous masses in the thoracic wall as metastases of a solitary mesothelioma of the diaphragm in a dog without involvement of ribs.

Metastases are rare and generally spread via the lymphatic system [11]. Nevertheless, cases with a suspected hematogenous dissemination of mesothelioma have been reported in dogs and cats [8, 12, 13]. In the case reported here, the involvement of subcutaneous tissue and ribs was a direct extension of the primary tumor to the chest wall rather than due to metastatic dissemination. Direct invasion was supported by the involvement of all structures of the chest wall, such as muscle layer, ribs and subcutaneous tissue. On the contrary, the multifocal pattern and the presence of intravascular emboli in the lung suggest a metastatic hematogenous spread rather than a direct invasion.

Bone destruction, in particular rib erosion, has not been reported in dogs with diffuse or solitary mesotheliomas. The differential diagnosis of lytic rib lesions includes primary or metastatic neoplasia, inflammatory and metabolic disease, and trauma. Primary tumors of the ribs mainly consist of osteosarcomas, chondrosarcomas, hemangiosarcomas, and fibrosarcomas, but osteosarcomas and hemangiosarcomas have been reported to develop bone metastasis as well [14]. In the present report, rib lesions could be attributed to a metastasis of the pelvic osteosarcoma or due to primary tumor.

The co-existence of mesothelioma and other malignancies appears relatively frequent in humans, and most of these tumors are carcinomas and adenocarcinomas [5, 15]. Exposure to asbestos has been closely related to the development of pleural mesotheliomas [16]. Risk factors for synchronous neoplasms such as male sex, older age, a positive smoking history, and a history of significant exposure to asbestos are risk factors for development of both types of neoplasms—either as a single and a concurrent neoplasm [17].

The simultaneous occurrence of mesothelioma and other primary tumors has not been described in dogs previously. The contribution of asbestos to canine mesothelioma remains unknown, although it is considered a risk factor [18, 19]. Other carcinogens may play an important role in the pathogenesis of these tumors, but a cause–effect relationship has not been identified.

## Conclusion

This case represents the first report of a diffuse pleural mesothelioma directly invading the thoracic wall in a dog with a concurrent osteosarcoma. Malignant mesotheliomas need to be considered a potentially locally invasive tumor in dogs and, therefore, should be included in the differential diagnosis when investigating a subcutaneous mass in the thoracic wall, especially in dogs with pleural effusion. Malignant mesotheliomas should be considered in the diagnostic workup of patients with lytic rib lesions.
